# Changes in Biochemical Characteristics and Activities of Ripening Associated Enzymes in Mango Fruit during the Storage at Different Temperatures

**DOI:** 10.1155/2014/232969

**Published:** 2014-07-22

**Authors:** Md. Anowar Hossain, Md. Masud Rana, Yoshinobu Kimura, Hairul Azman Roslan

**Affiliations:** ^1^Department of Biochemistry and Molecular Biology, University of Rajshahi, Rajshahi 6205, Bangladesh; ^2^Genetic Engineering Laboratory, Department of Molecular Biology, Faculty of Resource Science and Technology, Universiti Malaysia Sarawak, 94300 Kota Samarahan, Sarawak, Malaysia; ^3^Department of Biofunctional Chemistry, Graduate School of Natural Science and Technology, Okayama University, Okayama 700-8530, Japan

## Abstract

As a part of the study to explore the possible strategy for enhancing the shelf life of mango fruits, we investigated the changes in biochemical parameters and activities of ripening associated enzymes of Ashwina hybrid mangoes at 4-day regular intervals during storage at −10°C, 4°C, and 30 ± 1°C. Titratable acidity, vitamin C, starch content, and reducing sugar were higher at unripe state and gradually decreased with the increasing of storage time at all storage temperatures while phenol content, total soluble solid, total sugar, and nonreducing sugar contents gradually increased. The activities of amylase, *α*-mannosidase, *α*-glucosidase, and invertase increased sharply within first few days and decreased significantly in the later stage of ripening at 30 ± 1°C. Meanwhile polyphenol oxidase, *β*-galactosidase, and *β*-hexosaminidase predominantly increased significantly with the increasing days of storage till later stage of ripening. At −10°C and 4°C, the enzymes as well as carbohydrate contents of storage mango changed slightly up to 4 days and thereafter the enzyme became fully dormant. The results indicated that increase in storage temperature and time correlated with changes in biochemical parameters and activities of glycosidases suggested the suppression of *β*-galactosidase and *β*-hexosaminidase might enhance the shelf life of mango fruits.

## 1. Introduction

Mango (*Mangifera indica*, L) is the most important fruit of Bangladesh because of special characteristic flavor, pleasant aroma, taste, and nutritional value. Both ripe and raw fruits are also used to make a variety of products such as juice, chutney, and jelly. For these reasons, traditionally it is called the king of fruits in Bangladesh. Mango has medium calorific and high nutritional values especially carbohydrate [1]. Among the cultivated fruits in Bangladesh, it is the highest in terms of acres and second highest in terms of production. The cultivation area and the estimated production of mango per year in Bangladesh were 79066 acres and 1047849 metric tons, respectively [2]. Its local market sale generates about Tk. 52.39 billion. In a globalized economy, the control of fruit ripening is of strategic importance because excessive softening limits shelf life and storage. Softening plays an important role in cost implication, consumer acceptability, shelf life, and postharvest disease resistance. A considerable quantity (30–35%) of fruit turned waste through postharvesting process [3]. A survey was conducted on the softening of 18 mango varieties in 161 temporary storages of whole sellers and retailers at 20 spots in six districts of Bangladesh and found that the postharvest loss was 12.5% [4]. An effective strategy for enhancing the mango fruit shelf life is still lacking and, as a result, it is an economic loss for Bangladesh.

All biochemical and physiological changes during fruit ripening are driven by the coordinated expression of fruit ripening-related genes. These genes encode enzymes that participate directly in biochemical and physiological processes [5]. Generally, the ethylene production within the fruit activates many other enzymes resulting in physiological changes such as the change of color from green to red and the softening of the fruit. Excessive softening is associated with an increased expression of cell wall degrading enzymes acting upon protein and carbohydrates such as polygalacturonase, pectin methylesterase, *β*-galactosidase, and *β*-glucanase [6–8]. However, a number of studies have shown that polygalacturonase, pectin methylesterase, *β*-galactosidase, and *β*-glucanase suppression are not sufficient to significantly reduce the fruit softening [9–12]. It has been reported that *N*-glycan processing enzymes, beta-D-*N*-acetyl-hexosaminidase and *α*-D-mannosidase, are involved in tomato fruit ripening and suppression of these enzymes could enhance the shelf life of tomato fruits [13–15]. However, the roles of *N*-glycans in fruit ripening are still elusive and the detailed mechanism of fruit softening or ripening is not fully elucidated.

Earlier studies on mango fruit softening mainly focused on the postharvest physiology [16, 17], fruit flavors' volatiles [18], overall composition, and gross changes in total pectin during ripening [19]. However, detailed studies on the quantitative changes in biochemical parameters and activities of glycosidase enzymes involved during fruit ripening have not been carried out in mango fruits, particularly, the Ashwina variety. A change in storage temperature can affect the biochemical processes in fruits. Most biochemical characteristics of mango fruits such as total soluble solid, titratable acidity, starch, vitamin C, total sugar, reducing and nonreducing sugars, and total phenolic compounds could be affected by the postharvest storage temperatures.

Specifically, *N*-glycans molecules function to maintain the structural integrity of the cell walls. Cell wall glycans are chemically and conformationally diverse due to the complexity of their building blocks, the glycosyl residues, such as glucose, mannose, galactose, and xylose. Even *N*-glycan's composition varies in different cell and/or tissue types or even subdomains of a single cell wall [20]. Hence it might be speculated that common glycosidases may not be present in different fruits species due to availability of various glycosyl residues in the plant cell walls. Their concentrations may be different among the fruits species during ripening. The cell wall *N*-glycans degrading enzymes (*α*-mannosidase, *β*-hexosaminidase, *β*-galactosidase, and *α*-glucosidase) and other glycosidase enzymes (amylase and invertase) as well as polyphenol oxidase could be changed during the ripening process and affected by the storage temperatures. The biochemical parameters or glycosidases that are mostly related to mango fruit ripening or softening are still questionable. Therefore, the aim of the study was to investigate the changes in biochemical characteristics and activities of various glycosidases of Ashwina hybrid mangoes at 4-day regular intervals during storage at −10°C, 4°C, and 30 ± 1°C for an identification of biochemical parameters responsible for ripening process.

## 2. Materials and Methods

The experiments were carried out using the equipment available in the Protein Research Laboratory, Department of Biochemistry and Molecular Biology, University of Rajshahi, Bangladesh. Unless stated otherwise, all the reagents, synthetic and other substrates, used for performing the experiments were from Sigma-Aldrich and Carl-Roth GmbH, Germany.

### 2.1. Collection of Samples and Storage Procedure

Fresh mature green mangoes (Ashwina hybrid variety) were collected from Banesshar Bazar at Rajshahi, Bangladesh. Samples were sorted at the same maturity level and the pathogen-infected and/or mechanical damaged mangoes were discarded. The mangoes were stored at −10°C, 4°C, and 30 ± 1°C (room temperature) for a period of 12 days. Three mangoes were taken out from each group of samples on 4 days of regular interval of time such as on 0th, 4th, 8th, and 12th day and kept in −80°C for further analysis.

### 2.2. Preparation of Crude Extract

Fruits were cut into small pieces and homogenized by adding equal volume of extraction buffer (100 mM Tris-HCl, pH 7.0 with 0.25 M NaCl and 4 mM PMSF). The homogenized fruit was kept overnight at 4°C. After passing through four layers of muslin cloth, the extract was centrifuged at 10,000 ×g for 15 min to separate the debris. Supernatant was collected as crude extract. The various biochemical parameters were determined according to the following protocols.

### 2.3. Total Soluble Solids (TSS) and Titratable Acidity (TA)

TSS was determined according to the method as described by Mazumdar and Majumder [21] using digital bench refractometer (range 0–32%). An appropriate quantity of each sample was placed on the prism-plate of the refractometer and the reading appearing on the screen was directly recorded as total soluble solids (°Brix). TA was determined according to the method as described by Hortwitz [22]. Ten milliliters of crude extract was taken in a beaker and titrated with 0.1 N NaOH after adding 2-3 drops of phenolphthalein as an indicator. The percentage (%) of citric acid was calculated from the following formula:

(1) Titratable  Acidity(%) =Volume  of  0.12 N NaOH×Factor(0.0064)Volume  of  sample  used×100.

### 2.4. Vitamin C

Approximately 2 to 3 g of mango flesh was cut into small pieces and homogenized well with 20 mL of 3% metaphosphoric acid and filtered through double layers of muslin cloth. The filtrate was centrifuged at 3,000 ×g for 10 min and the clear supernatant was titrated with 2,6-dichlorophenol indophenol solution [23]. The amount of vitamin C in the extract was determined by comparing with the titration curve of standard vitamin C solution. Result was expressed in mg/100 g of fresh fruit.

### 2.5. Starch Content

The starch content of the mango flesh was determined by the anthrone method [24]. Two grams of mango was cut into small pieces and homogenized well with 20 mL water. It was then filtered through double layers of muslin cloth. Twice the volume of ethanol was added to the filtrate to precipitate the polysaccharide, mainly starch. After keeping overnight at 4°C, the precipitate was collected by centrifugation at 3,000 ×g for 15 min. The precipitate was heated to dryness and dissolved in 40 mL of 1 M HCl and then heated at 70°C for few minutes. It was then transferred to a volumetric flask and diluted to 100 mL with 1 M HCl. Aliquot of 1 mL of the extract of each sample was pipetted out into test tubes and 4 mL of the anthrone reagent was added to the each test tube and mixed well. The tubes were placed in a boiling water bath for 10 min and cooled. A blank reagent was prepared by using 1 mL of water and 4 mL of anthrone reagent in a test tube and treated similarly. The absorbance of the blue-green solution was measured at 680 nm in a colorimeter. The amount of starch present in mango flesh was calculated from the standard curve of different concentrations of glucose and expressed as g/100 g of fresh fruits.

### 2.6. Total Sugar (TS), Reducing Sugar (RS), Nonreducing Sugar (NRS), and Phenol Content (PC)

Two grams of mango flesh was cut into small pieces and plunged into 20 mL of boiling ethanol for 5–10 min. After cooling, it was crushed thoroughly in a mortar with a pestle. The homogenate was filtered through two layers of muslin cloth and refiltered through a Whatman no. 41 filter paper. The extract was evaporated to dryness over a steam bath and subsequently cooled. The residues were dissolved in 100 mL of distilled water and the resulting solution was used as flesh extract (sample stock) for the estimation of TS, RS, and PC.

#### 2.6.1. TS

TS content of mango was determined calorimetrically by the anthrone method [24]. Aliquot of 1 mL of the flesh extract was pipetted out into three test tubes and 4 mL of the anthrone reagent was added to each tube and mixed well. The test tubes were placed in a boiling water bath for 10 minutes. After cooling, the absorbance was measured at 680 nm against a blank reagent. The total sugar present in the sample tubes was determined from the standard curve prepared using different concentrations of glucose and expressed as g/100 g of mango flesh.

#### 2.6.2. RS

Reducing sugar content of the mango was determined by dinitrosalicylic acid (DNS) method [25]. Aliquot of 3 mL of the flesh extract was pipetted out into three test tubes and 3 mL of DNS reagent was added to each solution and mixed well. The test tubes were heated for 5 min in a boiling water bath. After developing the color, 1 mL of 40% Rochelle salt was added when the content of the tubes was still warm. The test tubes were then cooled under running tap water. The absorbance of the solution was measured at 575 nm in a colorimeter against a blank reagent. The amount of reducing sugar present in test tubes was calculated from the standard curve of glucose and expressed as g/100 g of mango flesh.

#### 2.6.3. NRS

Nonreducing content of the mango pulp was calculated from the following formula as described by Rahman et al. [26]:

(2) Nonreducing  sugar(%) =Total  sugar(%)−Reducing  sugar(%)×Factor(0.95).

#### 2.6.4. PC

PC was estimated by the Folinciocalteu reagent method [27]. Aliquot of 1 mL was pipetted out into three test tubes and 1 mL of Folinciocalteu reagent was added. After 3 min, 2 mL of 20 percent Na_2_CO_3_ solution was added to each tube. The contents were mixed thoroughly, and the tubes were placed in boiling water for 2 minutes. After cooling, the absorbance was measured at 650 nm against a blank reagent. A standard curve was plotted using different concentrations of catechol. From this standard curve, the concentration of phenols in the test sample was determined and expressed as mg/100 g of mango flesh.

### 2.7. Enzymatic Analysis

The activities of amylase, invertase, polyphenol oxidase, *β*-galactosidase, *α*-mannosidase, *α*-glucosidase, and *β*-hexosaminidase were assayed according to the following protocols.

#### 2.7.1. Amylase Activity Assay

Amylase activity was assayed according to the method described by Jayaraman [24]. One percent starch solution was used as substrate. The enzyme activity was measured by estimating the release of maltose calculated from the standard curve prepared with different concentrations of maltose. One unit of enzyme activity is defined as the amount of enzyme required to release 1 mg of maltose per minute at 37°C.

#### 2.7.2. Invertase Activity Assay

Invertase activity was assayed by the modified method as described by Mahadevan and Sridhar [28]. Sucrose was used as substrate. The invertase activity was measured by estimating the release of glucose calculated from the standard curve prepared with different concentrations of glucose. One unit of enzyme activity is defined as the amount of enzyme required to release 1 mg of glucose per minute at 37°C.

#### 2.7.3. Polyphenol Oxidase (PPO) Activity Assay

The PPO activity in mango fruit was measured as described by Mahadevan and Sridhar [28]. In this method, catechol was used as substrate. One unit of enzyme activity was defined as a change in absorbance of 0.001 per min per mL of enzyme extract. PPO activity was determined using a spectrophotometric method based on an initial rate of increase in absorbance at 495 nm. Aliquot of 2 mL of the crude extract and 3 mL of 0.1 M phosphate buffer pH 6.0 were pipetted out into a cuvette. The contents were mixed by inverting the cuvette and placed in a spectrophotometer set at 495 nm. Then the absorbance was adjusted to zero. The cuvette was removed and 1 mL of catechol was added and quickly mixed by inversion. The cuvette was placed in the spectrophotometer and the changes in absorbance at 495 nm were measured for 3 minutes.

#### 2.7.4. *β*-Galactosidase, *α*-Mannosidase, *α*-Glucosidase, and *β*-Hexosaminidase Activity Assay

Enzymatic activities of four glycosidases were assayed by estimating the release of *p*-nitrophenol according to the method described by Hossain et al. [15]. The 50 *μ*L of 100 *μ*M substrate (*p*-nitrophenyl-*α*-mannopyranoside/*p*-nitrophenyl-*α*-glucopyranoside/*p*-nitrophenyl-*β*-D-galactopyranoside/*p*-nitrophenyl-*β*-hexosaminide) was aliquoted into four separate Eppendorf tubes and 100 *μ*L of 100 mM phosphate buffer (pH 6.0) was added to each tube. A 50 *μ*L crude extract was then added to each tube and incubated at 37°C for an hour. To stop the reaction, 1.3 mL of glycine-NaOH buffer, pH 10.5, was added to each tube. The amount of *p*-nitrophenol released from substrates was measured by spectrophotometric method at the absorbance of 420 nm. One unit of enzyme activity is defined as the amount of enzyme required to release 1 nmol of *p*-nitrophenol per minute at 37°C.

## 3. Results and Discussion

### 3.1. Total Soluble Solid (TSS) and Titratable Acidity (TA)

**Figure 1:**
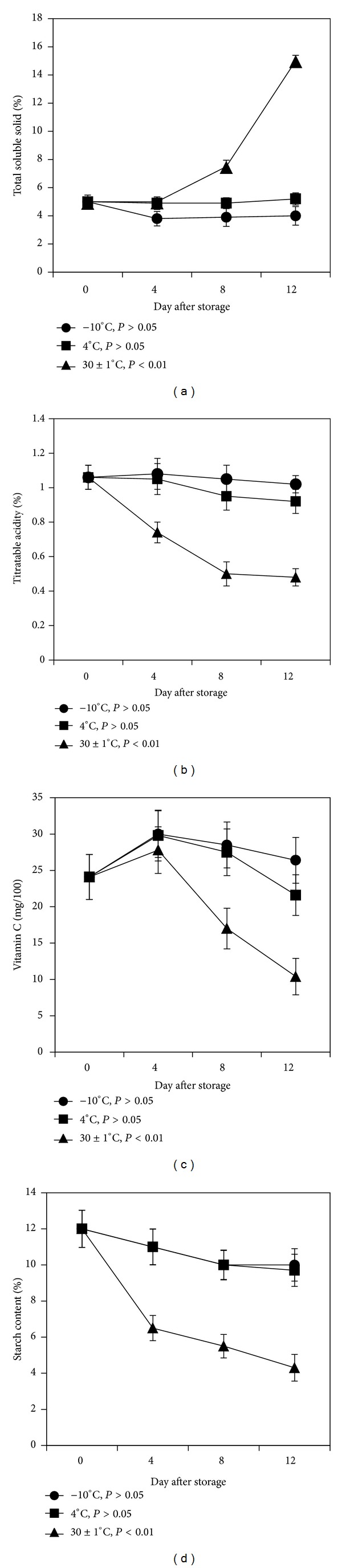
Changes in biochemical characteristics during ripening of mango fruits stored at −10, 4, and 30 ± 1°C for 12 days. (a) Total soluble solid; (b) titratable acidity; (c) vitamin C; (d) starch content. Each value represents the mean of three replicates and error bars represent standard deviation (SD).

TSS of fruits is a major quality parameter, which is correlated to the texture and composition [29]. The total soluble solids of mango fruit increased gradually with increasing the storage durations at ambient temperature. Figure 1(a) shows that TSS content of mango pulp did not increase at −10°C temperature but slightly decreased from 5.0 to 4.0 Brix till the 12th day of storage. It also remained at the same value till the 12th day at 4°C whereas TSS content did not increase at 30 ± 1°C until the 4th day of storage. However, TSS content increased significantly (*P* < 0.01) with the increasing days of storage till the 12th day. Figure 1(a) clearly shows that TSS content increased three times (5.0 to 15.0) from mature stage to ripening stage after 12 days of storage. This may be due to the degradation of cell walls and hydrolysis of starch to sucrose in the ripening stage.

The changes in TA are significantly affected by the rate of metabolism especially respiration, which consumed organic acid and thus declined acidity during the storage [30].  Figure 1(b) shows the changes in TA of mango fruit with the increase of storage time in different temperatures. TA decreased slightly (*P* > 0.05) from 1.06 to 1.02 (%) and 0.92(%) at −10°C and 4°C, respectively, after 12 days of storage. On the other hand, TA decreased significantly (*P* < 0.01) from 1.06 to 0.48 (%) with the increasing days of storage at 30 ± 1°C (Figure 1(b)). These results revealed that time and temperature are responsible for physicochemical changes of fruits and the major changes occur when fruits are stored for long time at high temperature. Low storage temperature (−10°C and 4°C) probably inhibited the activities of the enzymes to change the TSS and TA contents. Citric acid and malic acid are the main organic acids in “Keitt” mango, and decreases in titratable acidity with ripeness might be due to their utilization as substrates for respiration [31].

### 3.2. Vitamin C Content

Figure 1(c) shows that vitamin C content increased till the 4th day of storage at all storage temperatures observed and after that decreased with increasing days of storage till the 12th day. At −10°C, vitamin C content increased slightly from 24.1 to 26.4 mg/100 g fresh weight but at 4°C it decreased slightly to 21.6 mg/100 g fresh weight. On the other hand, at 30 ± 1°C vitamin C content decreased more sharply (*P* < 0.01) from 24.1 to 10.4 mg/100 g fresh weight. Findings clearly indicated that vitamin C content decreased with increasing days of storage during the ripening (Figure 1(c)) at ambient temperature. The reduction in vitamin C content of the fruit during ripening may be due to the susceptibility of ascorbic acid to oxidative destruction particularly at high ambient storage temperature [32].

### 3.3. Starch Content

The starch is the main carbohydrate present in mango fruits and the starch in mango pulp is hydrolyzed during ripening by amylase [33]. The starch content of mango pulp was found to decrease significantly (*P* < 0.01) from 12% to 4.3% with the increasing storage period at 30 ± 1°C till 12th day of storage (Figure 1(d)). On the other hand, the starch content decreased slightly till 4th day and remained unchanged even after 12 days of storage at −10°C and 4°C (*P* > 0.05). This was due to inactivation of mango amylase in lower temperature [34]. It was also reported that starch content remained unaltered in Fazli and Khirshapat varieties during the 10 days of storage at −5°C [26].

### 3.4. Total Sugar and Nonreducing and Reducing Sugar

**Figure 2:**
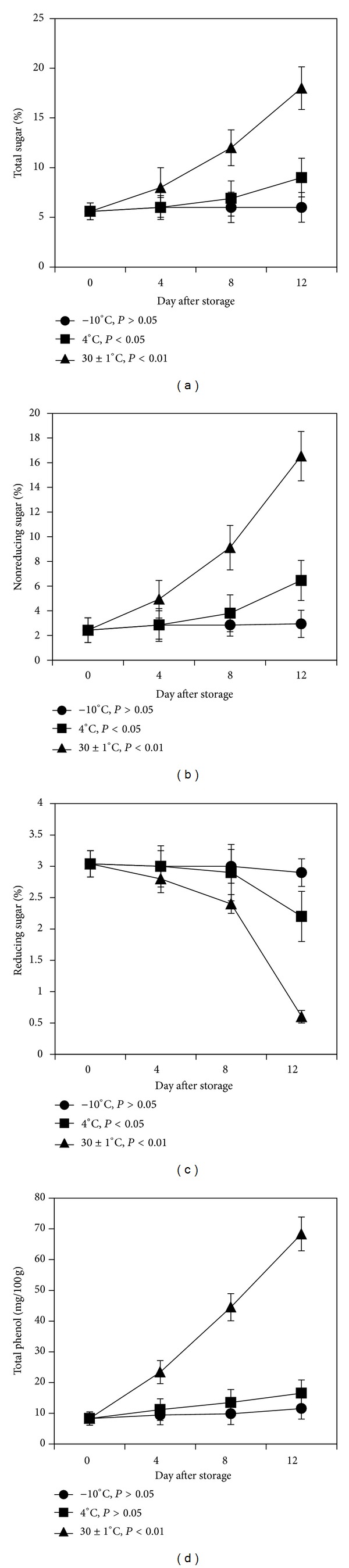
Changes in biochemical characteristics during ripening of mango fruits stored at −10, 4, and 30 ± 1°C for 12 days. (a) Total sugar; (b) nonreducing sugar; (c) reducing sugar; (d) total phenol content. Each value represents the mean of three replicates and error bars represent standard deviation (SD).

When mangoes were stored at 4°C and 30 ± 1°C, total sugar and nonreducing contents of mango pulp were found to be increased significantly over 12 days of storage (Figures 2(a) and 2(b)). At 30 ± 1°C, total sugar and nonreducing sugar contents increased by 3 and 7 times, respectively. On the other hand, when the mango was kept at −10°C, the total sugar and nonreducing sugar content increased slightly till 12th day. Rahman and his associates found similar trends of change in reducing and nonreducing sugar contents in Fazli and Khirshapat varieties after 10 days of storage at 4°C and 25°C [26]. When the mango was kept at −10°C, reducing sugar content of pulp remained unaltered at 3% till 8th day of storage and later decreased nonsignificantly (*P* > 0.05) till 12th day of storage (Figure 2(c)), while the reducing sugar contents were found to be decreased significantly at 4°C (*P* < 0.5) and 30 ± 1°C (*P* < 0.01) till 12th day of storage. Interestingly, reducing sugar contents decreased sharply from 3% to 0.6% over the 12 days of storage. The significant decrease in reducing sugar with increasing days of storage at 30 ± 1°C might be due to respiration and other energy consuming processes during the ripening of mango [35]. The respiration rates of mangoes were found to be faster at higher temperatures (28~40°C) and remained low and stable at low temperature (12°C) [36].

### 3.5. Total Phenol Content

It has been reported that total phenol content in Irwin mango stored at low temperature (5°C) increased till 20 days and after that decreased gradually [37]. Just after harvest initial total phenol content was observed at 83 mg/100 g fresh weight. The total phenol content increased slightly till 12th day reaching 115.4 mg/100 g and 165.3 mg/100 g at −10°C and 4°C, respectively. On the other hand, a remarkable increase (*P* < 0.01) in total phenol content from 83 mg/100 g to 683.6 mg/100 g was observed till 12th day of storage at 30 ± 1°C (Figure 2(d)). The results indicated that storage temperature and time remarkably affect the change in total phenol contents of mango fruits.

### 3.6. Activity of Amylase

**Figure 3:**
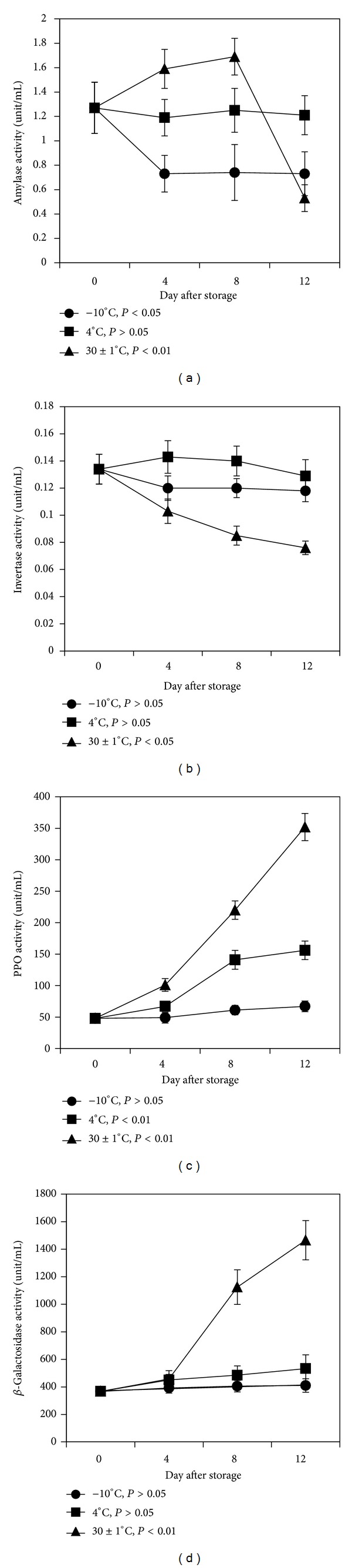
Changes in activity of different enzymes during ripening of mango fruits stored at −10, 4, and 30 ± 1°C for 12 days. (a) Amylase; (b) invertase; (c) polyphenol oxidase; (d) *β*-galactosidase. Each value represents the mean of three replicates and error bars represent standard deviation (SD).

Amylase is an enzyme that hydrolyses starch to yield monosaccharides.  Figure 3(a) shows that immediately after harvest the activity of amylase was found to be 1.27 unit/mL of crude extract. The activity of the enzyme remained almost constant from 0th day to 12th day of storage at 4°C whereas enzyme activity decreased to 0.73 unit/mL on 4th day of storage at −10°C and after that it remained unaltered. This result showed good correlation with the changes of starch content during storage at −10°C and 4°C (Figure 1(d)), where the starch content remained almost unchanged from 4th day to 12th day. At 30 ± 1°C, amylase activity was found to be increased in 1.69 unit/mL at the middle stage of ripening on 8th day and after that decreased abruptly to 0.53 unit/mL (Figure 3(a)). Rahman and his associates also found similar trends of amylase activity in Fazli and Khirshapat mangoes [26].

### 3.7. Activity of Invertase

Invertase is an enzyme that hydrolyzes sucrose to glucose and fructose, which increase sweetness of fruit. At −10°C the activity of invertase decreased slightly till 4th day and after that the activity of the enzyme remained almost constant about 0.120 unit/mL (Figure 3(b)); meanwhile at 4°C the activity of enzyme increased slightly till 4th day and subsequently the activity of the enzyme decreased nonsignificantly to 0.129 unit/mL with the increasing days of storage (Figure 3(b)). On the other hand, invertase activity decreased significantly (*P* < 0.01) to 0.076 unit/mL over 12 days of storage at 30 ± 1°C (Figure 3(b)). Ashwina variety is usually sour and not so sweet like Langra and Khirshapat variety. Hence, an accumulation of nonreducing sugar content (16.53%) correlates with the decrease in invertase activity at 30 ± 1°C (Figures 2(b) and 3(b)). The results indicated that invertase enzyme might not contribute to the ripening of mango fruits of the Ashwina variety.

### 3.8. Activity of Polyphenol Oxidase (PPO)

Figure 3(c) shows that the activity of PPO remained almost constant over 12 days of storage at −10°C, while it increased significantly from 4th day to 12th day of storage at 4°C. On the other hand, at 30 ± 1°C PPO the activity increased significantly (*P* < 0.01) from 48 to 352 units/mL till 12th day of storage. PPO activity of ripe mango was more than three times higher than that of unripe mango at 30 ± 1°C. A positive correlation was seen between the increasing PPO (Figure 3(c)) and total phenols during the 12 days of storage (Figure 2(d)). The results suggest that PPO, an enzyme related to browning process, can be used as a biomarker for fruit softening assay. Physiological disorders that cause discoloration of pulp and peel in mango fruit usually occur when they are stored at temperatures less than 13°C and PPO has been reported to be involved in this process [38]. It has also been shown that PPO and total phenolic compounds play important roles in the resistance of mango fruit to anthracnose disease and could be used as indicators to screen for mango cultivars that are more resistant to postharvest diseases [39].

### 3.9. Activity of *β*-Galactosidase

*β*-Galactosidase is a glycosidase that acts on short chain oligomers of *β*-galactose residues present either as homo-/heteropolysaccharides, glycoproteins, or glycolipids. At −10°C and 4°C, the activity of *β*-galactosidase increased slightly up to 4th day and after this period the activity of the enzyme remained almost constant till 12th day of storage (Figure 3(d)). On the other hand, at 30 ± 1°C the *β*-galactosidase activity increased gradually (*P* < 0.01) to 1466 unit/mL till 12th day of storage. The activity of *β*-galactosidase increased four times higher at 30 ± 1°C than that at −10°C and 4°C (Figure 3(d)). These results are supported by the experiments conducted by Rahman and his associates [26]. Among the glycosidases assayed, *β*-galactosidase was the most predominant enzyme found in Harumanis mango and the enzyme activity increased in parallel with increase in tissue softening during the storage [40]. The three isoforms of *β*-galactosidase found in Alphonso mango were able to degrade the endogenous substrate (arabinogalactan) and suggested a role in pectin dissolution [41]. This enzyme could be a key enzyme particularly responsible for cell wall modification and softening of the mango fruit during ripening.

### 3.10. Activity of *α*-Mannosidase

**Figure 4:**
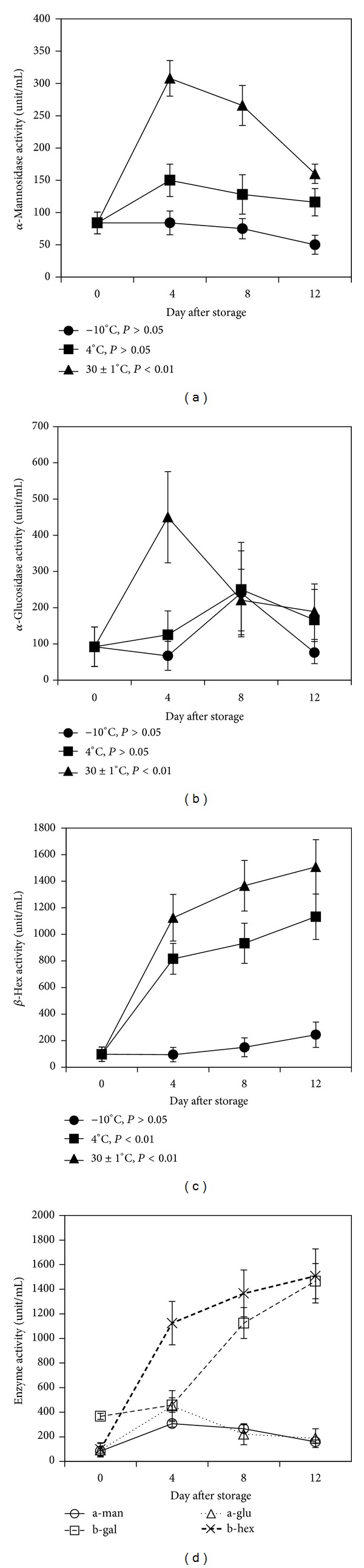
Changes in activity of different enzymes during ripening of mango fruits stored at −10, 4, and 30 ± 1°C for 12 days. (a) *α*-Mannosidase; (b) *α*-glucosidase; (c) *β*-hexosaminidase; (d) comparison of the activities of four glycosidases (*α*-mannosidase, *β*-galactosidase, *β*-hexosaminidase, and *α*-glucosidase) stored at 30 ± 1°C for 12 days. Each value represents the mean of three replicates and error bars represent standard deviation (SD).

*α*-Mannosidase is an enzyme that is involved in the modification of *N*-glycoproteins. At 30 ± 1°C, the activity of *α*-mannosidase increased rapidly (*P* < 0.01) up to 4th day and after that the activity of the enzyme gradually decreased (Figure 4(a)). On the other hand, at −10°C and 4°C the *α*-mannosidase activity increased slightly up to 4th day of storage. Further, the activity of *α*-mannosidase decreased gradually with the increasing days of storage till 12th day (Figure 4(a)). It has been reported previously that suppression of *α*-mannosidase extends the shelf life of tomato fruits [14]. Activity of *α*-mannosidase was observed in the early stage of ripening (within first few days) (Figure 4(a)), suggesting that *α*-mannosidase might play an important role in the onset of ripening of mango fruits.

### 3.11. Activity of *α*-Glucosidase

*α*-Glucosidase is an enzyme that acts on 1,4-alpha linkages and breaks down starch and disaccharides. At −10°C and 4°C, the activity of *α*-glucosidase increased slightly up to 8th day and after this period the activity of the enzyme decreased (Figure 4(b)). On the other hand, at 30 ± 1°C *α*-glucosidase activity increased rapidly (*P* < 0.01) up to 4th day and subsequently the activity decreased gradually with the increasing days of storage (Figure 4(b)). A correlation between the decrease in *α*-glucosidase activity (Figure 4(b)) and increase in polyphenolic compounds (Figure 2(d)) during the 12 days of storage was also observed. It has been reported that different polyphenolic components of soft fruits inhibit *α*-amylase and *α*-glucosidase [42]. Our results indicated that *α*-glucosidase or glucanase activity increased during the early stage of ripening but decreased in later stage and might play a role in the onset of ripening.

### 3.12. Activity of *β*-Hexosaminidase

*β*-Hexosaminidase is the member of GH family 20 that cleaves the terminal *N*-acetyl-D-hexosamine residues of glycoproteins and generates the paucimannosidic *N*-glycans. At −10°C the activity of *β*-hexosaminidase increased slightly up to 12th day of storage while the activity of enzyme at 4°C was found to increase gradually (*P* < 0.1) to 1133 unit/mL (Figure 4(c)). On the other hand, the activity of *β*-hexosaminidase at 30 ± 1°C increased sharply till 4th day and subsequently the activity increased significantly (*P* < 0.01) with the increasing days of storage and finally reached 1508 unit/mL on 12th day of storage (Figure 4(c)). The *β*-hexosaminidase activity of ripe mango at 30 ± 1°C was more than eight times higher than that of storage at −10°C and 4°C. Moreover, the *β*-hexosaminidase was found to be the second highest predominant enzyme among the tested enzymes during the fruit ripening. The result suggests that *β*-hexosaminidase might play a key role which is being responsible for mango fruit softening.It has been reported that suppression of the activities of *β*-hexosaminidase and *α*-mannosidase enhanced fruit shelf life of tomato fruit [14]. The molecular studies of two homologs of *β*- hexosaminidase suggested the involvement of these enzymes in the regulation of fruit softening in peach [43].

## 4. Significance of Changes in Biochemical Characteristics and Activities of Ripening Associated Enzymes

Textural softening during fruit ripening is of commercial importance as it directly dictates fruit shelf life, which is due to* in vivo* carbohydrate hydrolysis by respective carbohydrate hydrolases. The contents of vitamin C, total titratable acidity, total soluble solid, starch, and total phenol changed nonsignificantly at −10°C and 4°C temperature due to inactivation of enzymes. The total soluble solid and total phenol content increased whereas titratable acidity, starch, and vitamin C decreased significantly at 30 ± 1°C. All sugar contents (total sugar, reducing sugar, and nonreducing sugar) changed nonsignificantly at −10°C but they either increased or decreased significantly at 4°C and 30 ± 1°C. The activities of all the enzymes examined in the study were found to be higher in mango pulp at 30 ± 1°C as compared to those stored in 4°C or −10°C. Amylase, *α*-glucosidase, and *α*-mannosidase were found to be increased in the middle stage of ripening. The activities of these enzymes seemed to indicate the roles played in the onset of fruit ripening process. At 30 ± 1°C, the *β*-hexosaminidase and *β*-galactosidase had higher activities than other glycosidase enzymes tested in this study (Figure 4(d)). The prominent enzymes were found to be polyphenol oxidase, *β*-hexosaminidase, and *β*-galactosidase which gradually increased significantly from onset of ripening to later stage of ripening when stored at 30 ± 1°C. It is not clear whether the polyphenol oxidase (PPO) plays any role in fruit ripening although it predominantly increased with fruit softness. Lowering the storage temperature to below the ambient temperature (<30 ± 1°C) may extend the shelf life of mango fruits. The results indicated that *β*-galactosidase and *β*-hexoseaminidase are predominantly associated with textural softening of mango fruits, which may pave the way for finding the effective strategy or approach for enhancing the shelf life of mango fruits to minimize the postharvest loss. The suppression of the expression of these enzymes might be an effective strategy for enhancing the shelf life of mango fruits. The generation of transgenic plant, in which galactosidase and *β*-hexoseaminidase are suppressed or overexpressed, is underway in our laboratory which will be published elsewhere in future.
